# Diagnostic performance and image quality of iterative model-based reconstruction of coronary CT angiography using 100 kVp for heavily calcified coronary vessels

**DOI:** 10.1371/journal.pone.0222315

**Published:** 2019-09-10

**Authors:** Junwoo Kim, Bon Seung Goo, Young-Seok Cho, Tae-Jin Youn, Dong Jun Choi, Amar Dhanantwari, Mani Vembar, Eun Ju Chun

**Affiliations:** 1 Department of Radiology, Seoul National University Bundang Hospital, Sungnam, Korea; 2 Department of Internal Medicine, Seoul National University Bundang Hospital, Sungnam, Korea; 3 CT/AMI Clinical Science, Philips Healthcare, Highland Heights, OH, United States of America; Universita degli Studi Magna Graecia di Catanzaro, ITALY

## Abstract

**Objectives:**

To evaluate the diagnostic performance and image quality of an iterative model-based reconstruction (IMR) using a 100-kVp protocol for the assessment of heavily calcified coronary vessels, compared to those of filtered back projection (FBP) and hybrid iterative technique (iDose^4^), and also compared to those of IMR with standard 120 kVp protocol.

**Methods:**

Among patients with Agatston scores ≥ 400 who had undergone both coronary CT angiography (CCTA) and invasive coronary angiography (ICA), age- and sex-matched patients with body mass index < 30 were retrospectively enrolled from CCTA with low-kVp protocol (100 kVp, n = 30) and with standard-kVp protocol (120 kVp, n = 30). Image data were all reconstructed with FBP, iDose^4^, and IMR. In each dataset, the objective and subjective image quality, and diagnostic accuracy (> 50% in luminal reduction as compared with ICA) were assessed.

**Results:**

IMR showed better objective and subjective image quality than FBP and iDose^4^ in both 100 kVp and 120 kVp groups (all p < 0.05). IMR showed a significantly improved all diagnostic performance compared with FBP (p < 0.05). Compared with iDose^4^, IMR significantly improved positive predictive value (85.0% vs. 80.5%; p < 0.05). There was no significant difference in image quality and diagnostic performance using IMR between the 100 kVp and 120 kVp groups.

**Conclusions:**

100 kVp IMR may be useful for the assessment of heavily calcified coronary vessels, providing better diagnostic performance than FBP or iDose^4^ at the same dose, while maintaining similar diagnostic accuracy to 120 kVp IMR.

## Introduction

Coronary computed tomography angiography (CCTA) has emerged as a robust non-invasive method with high sensitivity and negative predictive value to rule out coronary artery stenosis [1, 2]. Despite the advancements in CT techniques, evaluation of heavily calcified coronary vessels still remains challenging due to the presence of blooming artifacts [3]. When a dense, calcified plaque is located adjacent to a structure with very low density, calcium blooming may lead to overestimation of luminal stenosis [4]. It accounts for false positivity, it impacts specificity and diagnostic accuracy [1, 5]. Therefore, some institutions insist on avoid performing CCTA in patients with CACS > 1000 [6]. Although the use of high energy (kVp) imaging can reduce blooming artifacts, it results in an increased radiation exposure to the patients, which is very challenging in the era of reducing radiation dose as low as possible.

Over the past several years, iterative reconstruction (IR) techniques–aimed at reducing noise and thus the radiation exposure received by patients–have been used in the clinical setting. It has been reported that the use of hybrid type iterative techniques (hybrid-IR) enable a reduction in the radiation dose while maintaining image quality compared with the filtered back projection (FBP) technique [7, 8]. Moreover, Renker et al. [9] reported that the IR technique has the potential to improve diagnostic accuracy of CCTA for calcified coronary vessels because it can reduce calcium blooming.

Recently, iterative model-based reconstruction algorithm (IMR) has been introduced. IMR approaches reconstruction by optimizing the algorithm of cost function while considering the data statistics, image statistics, and system models. It matches the reconstructed image to the acquired data iteratively to produce high spatial resolution images with low noise [10, 11]. However, there have been no reports regarding the diagnostic accuracy of CCTA with the use of IMR reconstruction to evaluate heavily calcified coronary vessels, especially with respect to the feasibility of IMR using a low-kVp protocol.

In this study, we investigated whether IMR via the low-kVp protocol is useful for the assessment of heavily calcified coronary vessels. To do this, we compared the image quality and diagnostic performance of three reconstruction methods (FBP, hybrid-IR [iDose^4^, Philips Healthcare, Cleveland OH, USA], IMR) on standard-kVp (120 kVp) and low-kVp (100 kVp) with age- and sex-matched.

## Materials and methods

### Study population

The local institutional review board (Seoul National University Bundang Hospital) approved this retrospective study and a waiver of the requirement for informed consent. IMR has been introduced in our institution in October 2013. After several months of testing, in April 2014, the standardized CCTA protocol has changed to 100 kVp from 120 kVp for all patients with a BMI of less than 30. Among patients with suspected coronary artery disease (CAD) according to the CCTA data registry between October 2013 and May 2015, we retrospectively enrolled those that matched the following criteria: (1) heavily calcified coronary vessels with an Agatston score of ≥ 400; and (2) history of receiving invasive coronary angiography (ICA) within two months after CCTA scan. From this group, we consecutively allocated each individual with a BMI of less than 30 into the standard kVp group where 120 kVp was applied (n = 30) or the low kVp group where 100 kVp was applied (n = 30) by matching of age and gender.

Among them, patients with previous stent or coronary artery bypass graft were excluded. Finally, a total of 60 patients (44 males; mean age, 69.3 ± 8.9 years; mean age of male, 67.8 ± 9.4 years; of female, 73.4 ± 5.3 years) with heavily calcified coronary vessels were enrolled in this study.

### Coronary CT angiography protocol

Coronary artery calcium scoring (CACS) and CCTA were performed on a 256-slice CT scanner (Brilliance-iCT; Philips Healthcare, Cleveland, OH) with a 128 x 0.625-mm detector collimation, 272-millisecond tube rotation time. Prior to contrast injection, CACS was performed using a prospective electrocardiographically (ECG)-triggered acquisition technique with 120-kV tube voltage, 55-mAs tube current, and 2.5-mm scan thickness. Agatston score was calculated using a threshold of 130 Hounsfield units (HU) [12].

Patients with a baseline heart rate > 70 beats/min received 10 ~ 40 mg of intravenous Esmolol (Jeil Pharm, Seoul, Korea) before the CT scan to lower and stabilize the heart rate. For those who did not have contraindications to nitroglycerin, a dose of 0.6 mg was administered sublingually immediately before contrast injection [13]. To minimize the radiation dose, CCTA technique was obtained 100 kVp with ECG-dose modulation technique. The traditional retrospective ECG gating with a default use of ECG dependent tube current modulation was routinely used in all patients for an analysis of cardiac function. During CCTA acquisition, a bolus of 80 ml Iomeprol (Iomeron 400, Bracco, Milan, Italy) was injected intravenously (4 ml/s), followed by a 50-ml saline chaser.

### CT reconstruction

From the CCTA raw data for each patient, the images were reconstructed with a section thickness of 0.9 mm and an interval of 0.45 mm. All images were reconstructed with three different reconstruction algorithms: FBP; hybrid-IR (iDose^4^; Philips Healthcare, Cleveland, OH) set to level 4; and finally, knowledge-based IR (IMR; Philips Healthcare, Cleveland, OH) with cardiac routine setting and level 2. Post-processing and reconstruction were performed for qualitative evaluation with curved planar reformatted images of each vessel, using commercially available software (EBW, V4.5, Philips Medical systems, Cleveland, OH, USA). All images were anonymized, and visible scan parameters were removed.

### CCTA image analysis

**Fig 1:**
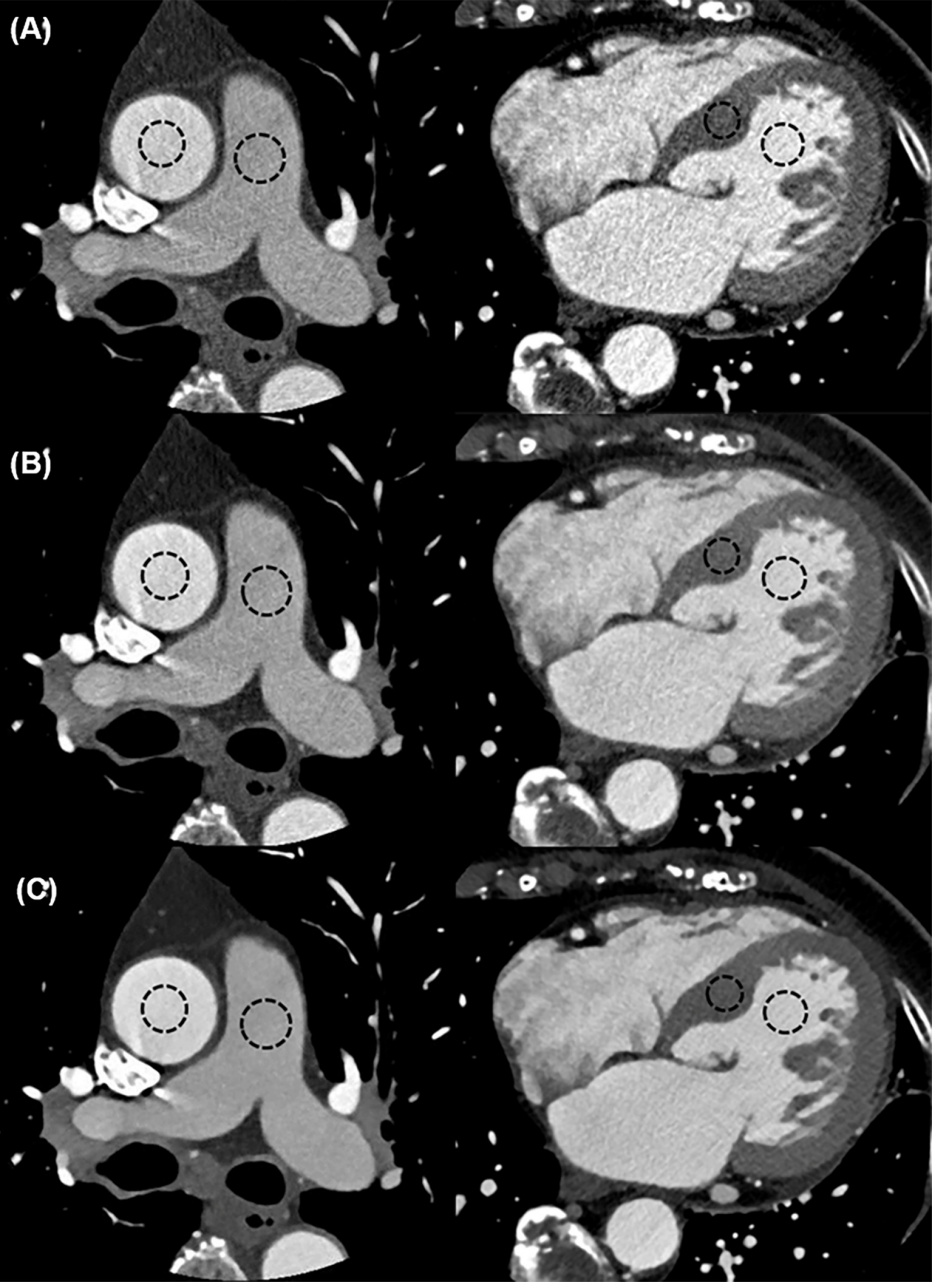
Objective image quality of each reconstruction method. Image noise was defined as the standard deviation (SD) of the measured attenuation within circular region of interest (ROI) in the ascending aorta, main pulmonary artery, interventricular septum, and left ventricular cavity. (A) FBP, (B) iDose^4^, (C) IMR.

**Fig 2:**
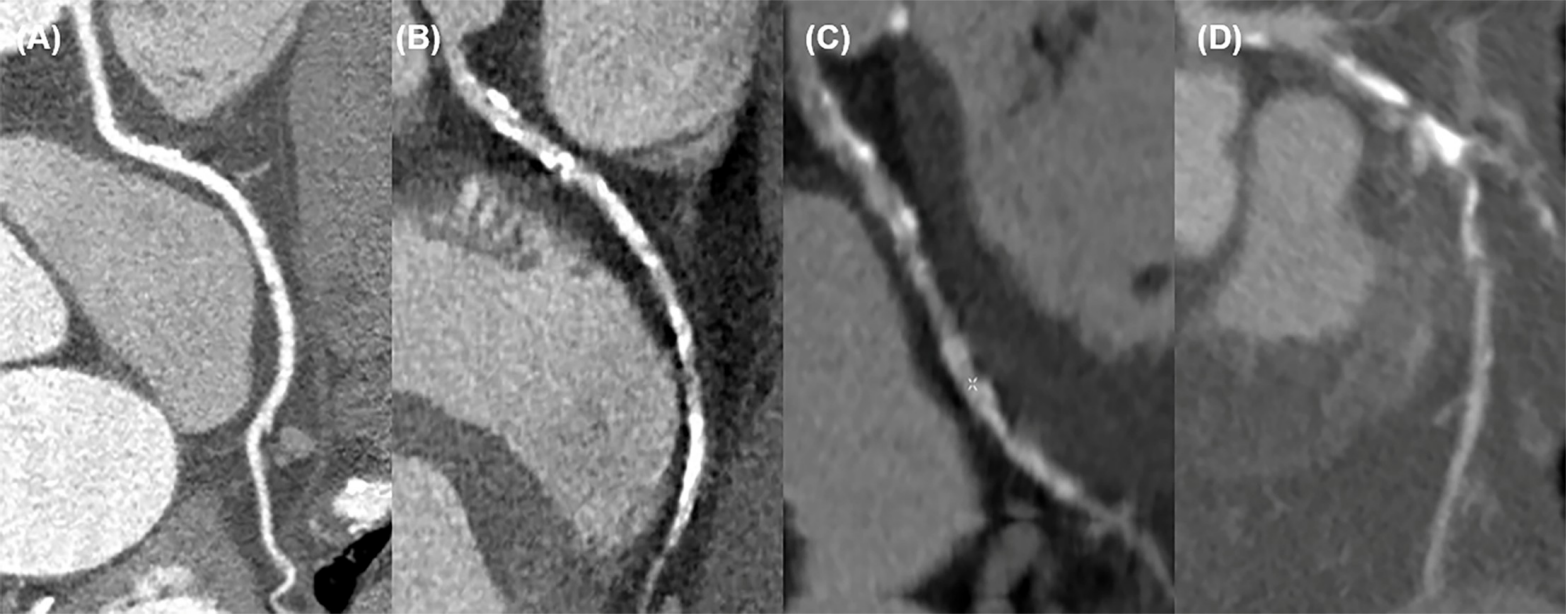
Subjective image quality. (A) Excellent denoted smooth and clear vessel countour without any noise or artifact, providing useful diagnostic information. (B) Good denoted slightly noisy or artefactual image, but with clear vessel contour, providing sufficient diagnostic information. (C) Fair denoted noisy and artefactual image with partial obscurity in vessel countour, providing acceptable diagnostic information. (D) Poor denoted a very noisy and artefactual image, providing insufficient diagnostic information.

Both objective and subjective image qualities were compared between groups. Objective image quality was a measurement of image noise by one observer (B.S.G. 9 years of CCTA imaging experience). The image noise was defined as the standard deviation (SD) of the measured attenuation within a circular region of interest (ROI) in the ascending aorta, main pulmonary artery, interventricular septum, and left ventricular cavity (Fig 1). The size of ROI took into account, as much as possible, the anatomical differences between patients; however, it was kept at a constant size between the different reconstruction approaches. For subjective image quality, each coronary vessel was rated according to the following 4-point grading scale: 4 ‘excellent’ represented image with minimum noise and no artifacts, with smooth and clear contours, and useful diagnostic information; 3 ‘good’ represented image with slight (but acceptable) noise or artifacts, with clear contours, and sufficient diagnostic information; 2 ‘fair’ represented noisy image and artefactual, with partially obscured contours, but with acceptable diagnostic information; and 1 ‘poor’ represented very noisy image and artefactual, and insufficient information for diagnosis (Fig 2).

For diagnostic accuracy, stenosis severity was estimated using the contrast-enhanced portion of the coronary lumen semi-automatically traced using a commercially available workstation (IntelliSpace Portal (ISP) Version 8, Philips Healthcare, Best, The Netherlands) at the maximal stenotic site and compared with the mean value at the proximal and distal reference sites. Stenosis degree was reported at 10% intervals, and stenosis > 50% was deemed significant. The analysis was performed on per segment-basis and also on a per patient-basis. The segment-based analysis included all segments according to American Heart Association 16-segment model [14], except for the fine vessel with a diameter of less than 1mm. In terms of the patient-based analysis, a true positive was defined as the presence of at least one positive segment without any false-positive segment, regardless of location.

Images reconstructed with FBP, iDose^4^, and IMR were intermixed, and the subjective image quality and degree of stenosis for each data set were blindly assessed by two experienced cardiac radiologists (J.K. 3 years; E.J.C. 10 years for CCTA imaging) without any clinical information. After independent evaluations, a consensus was made to obtain the final CCTA diagnosis because one unified result had to be made available for comparison of diagnostic accuracy among three different reconstructions.

### Invasive coronary angiography

ICA was performed using 5-French high-flow Judkins catheters (Cordis, Miami, FL, USA), and images were acquired in multiple projections. Two experienced cardiologists (Y.S.C. 10 years; T.J.Y. 11 years for ICA), who were blinded to the CCTA results, analyzed the coronary angiograms using a validated quantitative coronary angiographic system (Allura DCI program, Philips Medical Systems, Best, The Netherlands) for determining the degree of coronary artery stenosis according to the same 16-segment model. The severity of coronary stenosis was quantified in two orthogonal views, and obstructive CAD was classified as significant if the lumen diameter reduction was > 50%.

### Statistical analysis

Continuous variables were expressed as the means ± standard deviation (SD); categorical variables were presented as absolute values and percentages. Inter-observer agreement for identifying image quality and stenosis degree were calculated using κ statistics in the following manner: poor (< 0.20), fair (0.20–0.40), moderate (0.41–0.60), good (0.61–0.80), and excellent agreement (0.81–1.00).

The subjective and objective image quality of IMR were compared with those of FBP and iDose^4^ on each group (100 kVp and 120 kVp) and were one-way ANOVA with Bonferroni’s post-hoc analysis. To detect the significant obstructive lesions, we determined the diagnostic performances of CCTA in each group by two analyses–segment-based analysis and patient-based analysis–using ICA as the reference standard. Sensitivity, specificity, positive predictive value (PPV), negative predictive value (NPV), and accuracy of each three reconstruction techniques in each 100 kVp and 120 kVp were compared using exact McNemar test.

Finally, for comparison of image quality of IMR between 100 kVp and 120 kVp were used by paired t-test, while diagnostic performance of IMR between 100 kVp and 120 kVp were compared using Fisher’s exact test. P values of < 0.05 were considered statistically significant. Statistical analyses were performed using SPSS 20.0 (SPSS, Chicago, Illinois, USA).

## Results

### Baseline characteristics

All 60 CCTA reconstructions were successfully completed and considered to be the diagnostic image quality. For segment-based analysis, 454 segments in the 100 kVp group and 456 segments in the 120 kVp group were finally analyzed after excluding segments with a diameter of less than 1mm. Patient demographics and CCTA characteristics are summarized in Table 1. The average Agatston calcium score was 1308.7, reflecting advanced atherosclerosis. There were no statistical differences in all baseline characteristics, including various clinical risk factors and the mean heart rate between the two groups (all p > 0.05). In the 100 kVp group, the mean CTDI_vol_ was 35.6 ± 5.6 mGy and the mean DLP was 682.6 ± 152.6 mGy·cm, which were significantly lower than those in the 120 kVp group (all p < 0.001). Although the scan range in the 100kVp group was significantly higher than that in the 120 kVp group (141.5 ±24.2 cm vs. 127.5 ± 11.2, p = 0.006), the mean effective radiation dose was significantly lower in the 100 kVp group than in the 120 kVp group (9.5 ± 2.1 mSv vs. 12.9 ± 2.6, p < 0.001).

**Table 1:** Patient demographics and scan parameters.

Parameter	Total (n = 60)	Low-kVp group (n = 30)	Standard-kVp group (n = 30)	p-value
Age (years), mean±SD	69.3±8.8 (range 43–81)	68.2± 9.6 (range 43–80)	70.4 ± 7.9 (range 44–81)	0.337
Male	44 (73.3%)	23 (76.7%)	21 (70.0%)	0.771
Body mass index (kg/m^2^)	24.3 ±3.2 (range 16.8–31.5)	24.3 ± 2.9 (range 16.8–31.5)	24.2 ± 3.5 (range 17.0–31.2)	0.900
Risk factors for coronary artery disease				
Diabetes	25 (41.7%)	13 (43.3%)	12 (40.0%)	1.000
Hypertension	47 (78.3%)	24 (80.0%)	23 (76.7%)	1.000
Current smoker	12 (20.0%)	8 (26.7%)	4 (13.3%)	0.333
Ex-smoker	21 (35.0%)	10 (33.3%)	11 (36.7%)	1.000
Hyperlipidemia	17 (28.3%)	10 (33.3%)	7 (23.3%)	0.567
Family history of previous CHD	7 (11.7%)	3 (10.0%)	4 (13.3%)	1.000
Medication				
Statin	12 (20.0%)	5 (16.7%)	7 (23.3%)	0.748
Aspirin	20 (33.3%)	7 (23.3%)	13 (43.3%)	0.170
Heart rate (beats/min)	64.0 ± 11.9 (range; 34–97)	63.9 ± 11.7 (range; 38–90)	64.2 ± 12.4 (range; 34–97)	
NSR with HR < 75/min	42 (70.0%)	22 (73.3%)	20 (66.7%)	0.779
HR > 75/min	11 (18.3%)	5 (16.7%)	6 (20.0%)	1.000
Arrhythmia or non-NSR	9 (15.0%)	4 (13.3%)	5 (16.7%)	1.000
Agatston score	1308.7 ± 903.6	1376.3 ± 873.5 (range; 427.7–3699.6)	1241.1 ± 942.8 (range; 412.8–3985.9)	0.567
400–1000	30 (50.0%)	12 (40.0%)	18 (60.0%)	0.196
> 1000	30 (50.0%)	18 (60.0%)	12 (40.0%)	
kVp	-	100	120	
Tube current-time product (mAs)	-	920.3 ± 96.5	812.0 ± 141.2	0.001
CT dose index volume (mGy)	-	35.6 ± 5.6	52.2 ± 9.1	<0.001
Dose-length product (mGy·cm)	-	682.6 ± 152.6	925.4 ± 188.3	<0.001
Scan range (cm)	-	141.5 ±24.2	127.5 ± 11.2	0.006
Effective radiation dose (mSv)	-	9.5 ± 2.1	12.9 ± 2.6	<0.001

Note—SD, standard deviation; CHD, coronary heart disease; HR, heart rate; NSR, normal sinus rhythm, kVp, peak kilovoltage

### Image quality analysis

#### Comparison of objective image quality

Table 2 summarizes the objective image quality assessment in each 100 kVp group and 120 kVp group. The mean attenuation in the ascending aorta, pulmonary artery, LV cavity and septum was not significantly different among three reconstruction methods in both 100 kVp and 120 kVp protocols. However, the image noise using IMR at all the anatomical locations was significantly lower than that using FBP and iDose^4^ (all p < 0.001). Bonferroni post-test showed that the image noise was significantly different among three different reconstructions, while the mean attenuations at all locations did not different among three reconstruction methods.

**Table 2:** Comparison of objective image quality of CCTA using three different reconstruction methods in each of the 100 kVp and 120 kVp protocols.

	HU^1)^			SD^1)^^,^ ^2)^		
ROI	FBP	iDose4	IMR	FBP	iDose4	IMR
***120 kVp (n = 30)***						
Ascending aorta	414.7 ± 71.9	415.6 ± 72.3	415.2 ± 72.4	58.7 ± 12.1	40.6 ± 8.4	16.5 ± 2.6
Pulmonary artery	341.3 ± 75.1	342.2 ± 75.1	341.9 ± 74.6	60.2 ± 13.7	41.5 ± 9.8	16.6 ± 4.9
Left ventricle cavity	380.4 ± 66.5	379.6 ± 68.1	380.0 ± 66.9	59.0 ± 10.0	39.4 ± 7.7	15.4 ± 2.5
Left ventricle septum	106.6 ± 17.1	115.1 ± 17.8	106.4 ± 16.5	59.4 ± 11.6	39.5 ± 8.6	16.1 ± 3.2
***100 kVp (n = 30)***						
Ascending aorta	475.7 ± 68.6	476.1 ± 69.4	461.1 ± 68.4	42.4 ± 6.3	29.2 ± 5.7	17.4 ± 3.5
Pulmonary artery	392.2 ± 71.4	395.1 ± 73.6	393.1 ± 79.4	44.1 ± 7.7	30.5 ± 7.2	17.5 ± 3.8
Left ventricle cavity	455.2 ± 67.3	457.9 ± 67.9	450.8 ± 66.4	50.1 ± 10.9	32.4 ± 7.7	19.0 ± 4.1
Left ventricle septum	134.0 ± 27.8	135.8 ± 28.9	131.0 ± 28.1	48.0 ± 9.8	31.8 ± 6.4	19.1 ± 5.1

Data are means ± standard deviations of the regions of interest.

Note–HU, Hounsefield unit; SD, standard deviation; ROI, region of interest; FBP, filtered back projection; iDose4, hybrid-iterative reconstruction set to level 4; IMR, iterative model reconstruction

^1)^ Statistical significance among three reconstruction methods was one-way ANOVA with Bonferroni post-hoc nalysis.

^2)^ Significantly different among three different reconstructions using Bonferroni post-test

#### Comparison of subjective image quality

Table 3 summarizes the subjective image quality assessment in each 100kVp group and 120kVp group. The visual scores of all coronary segments using IMR were significantly higher than those using FBP and iDose^4^ (all p < 0.05) in each 100 kVp and 120 kVp groups. The inter-reader agreement of subjective image quality of each vessels were good using FBP (ĸ = 0.61), iDose^4^ (ĸ = 0.68) and IMR (ĸ = 0.70) in 100 kVp group, and using FBP (ĸ = 0.68), iDose^4^ (ĸ = 0.72) and IMR (ĸ = 0.77) in 120 kVp group.

**Table 3:** Comparison of subjective image quality of CCTA using three different reconstruction methods in each of the 100 kVp and 120 kVp protocols.

	Image quality grading score			
	FBP	iDose^4^	IMR	p-value
***120 kVp (n = 30)***				
LAD	3.20 ± 0.71	3.57 ± 0.57	3.73 ± 0.45	<0.05*
LCx	3.50 ± 0.63	3.60 ± 0.50	3.80 ± 0.41	<0.05*
RCA	3.27 ± 0.79	3.59 ± 0.57	3.78 ± 0.42	<0.05*
Total	3.32 ± 0.72	3.58 ± 0.54	3.77 ± 0.43	<0.05*
***100 kVp (n = 30)***				
LAD	3.20 ± 0.89	3.43 ± 0.86	3.67 ± 0.66	<0.05*
LCx	3.13 ± 0.90	3.38 ± 0.78	3.55 ± 0.69	<0.05*
RCA	3.33 ± 0.71	3.37 ± 0.77	3.57 ± 0.68	<0.05*
Total	3.22 ± 0.83	3.39 ± 0.79	3.66 ± 0.60	<0.05*

Note–FBP, filtered back projection; iDose4, hybrid-iterative reconstruction set to level 4; IMR, iterative model reconstruction; LAD, left anterior descending artery; LCX, left circumflex artery; RCA, right coronary artery

*P < 0.05 means significant statistically significant.

### Diagnostic accuracy

Per-patient and per-segment diagnostic performances using three different reconstruction protocols of each 120 kVp and 100 kVp protocols are summarized in Table 4.

**Table 4:** Diagnostic accuracy of CCTA using three different reconstruction methods in each of the 100 kVp and 120 kVp protocols.

	Per patient			Per segment		
	FBP	iDose^4^	IMR	FBP	iDose^4^	IMR
***120 kVp (n = 30)***						
Sensitivity	96.2%	100%	100%	82.6%	87.5%	90.3%*
Specificity	50.0%	60.0%	60.0%	90.9%	94.2%	95.6%*
Positive predictive value	92.6%	92.6%	92.6%	75.4%	83.1%	87.2%*†
Negative predictive value	66.7%	100%	100%	93.9%	95.9%	96.8%*
Diagnostic accuracy	90.0%	93.3%	93.3%	88.8%	92.5%	94.3%*†
***100 kVp (n = 30)***						
Sensitivity	96.2%	96.2%	96.2%	80.2%	85.6%	89.2%*
Specificity	25.0%	50.0%	75.0%	89.5%	93.3%	95.0%*
Positive predictive value	89.3%	92.6%	96.2%	71.2%	80.5%	85.3%*†
Negative predictive value	50.0%	66.7%	75.0%	93.3%	95.2%	96.4%*
Diagnostic accuracy	86.7%	90.0%	93.3%	87.2%	91.4%	93.6%*†

Note—FBP, filtered back projection; iDose4, hybrid-iterative reconstruction set to level 4; IMR, iterative model reconstruction

* p<0.05, statistical significance of diagnostic performance using by exact McNemar test between IMR and FBP

† p<0.05, statistical significance of diagnostic performance using by exact McNemar test between IMR and iDose^4^

#### Per-patient analysis

In the 120 kVp protocol, per-patient based sensitivity, specificity, PPV, NPV, and diagnostic accuracy of IMR reconstruction were 100%, 60.0%, 92.6%, 100%, and 93.3%, respectively. These per-patient diagnostic performances were not significantly different from those using FBP (96.2%, 50%, 92.6%, 66.7% and 90.0% respectively) and iDose^4^ (100%, 60.0%, 92.6%, 100%, and 93.3%, respectively).

In 100 kVp protocol, per-patient based sensitivity, specificity, PPV, NPV, and diagnostic accuracy of IMR reconstruction were 96.2%, 75.0%, 96.2%, 75.0%, and 93.3%, respectively. The diagnostic performances using IMR were not significantly different from those using FBP (96.2%, 25%, 89.3%, 50% and 86.7% respectively) and iDose^4^ (96.2%, 50%, 92.6%, 66.7% and 90%, respectively).

#### Per-segment analysis

In 120 kVp protocol, per-segment based sensitivity, specificity, PPV, NPV, and diagnostic accuracy of IMR reconstruction were 90.3%, 95.6%, 87.2%, 96.8% and 94.3%, respectively. These were significantly higher than those of FBP (82.6%, 90.9%, 75.4%, 93.9%, and 88.8%, respectively) (p<0.05 for all). While IMR showed a significant improvement over iDose^4^ with respect to PPV (87.2% versus 83.1% respectively, p< 0.05) and accuracy (94.3% versus 92.5% respectively, p < 0.05), there were no significant differences in the performance otherwise.

**Fig 3:**
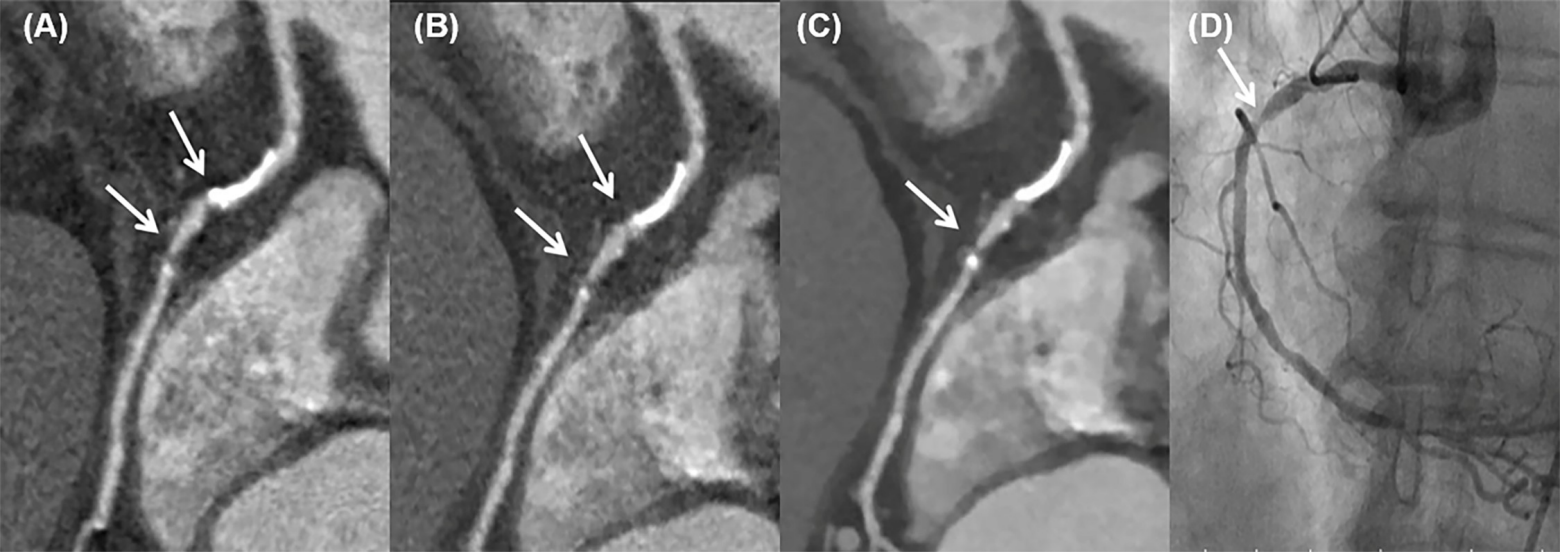
A 48-year-old female with high calcium score, of greater than 800. Curved MPR reformatted RCA with filter back projection image (A) and iDose^4^ image (B) shows two significant obstructive lesions are seeen at mid RCA (arrows). However, IMR image (C) shows that the lesion in the proximal portion with clacification was a false lesion due to blooming artifact and only the lesion in the distal portion was a significant obstructive lesion. Invasive coronary angioraphy (D) confirmed the presence of one lesion as shown in the IMR image.

In 100 kVp protocol, sensitivity, specificity, PPV, NPV, and diagnostic accuracy of IMR reconstruction were 89.2%, 95.0%, 85.3%, 96.4%, and 93.6% respectively, which were also significantly higher than those of FBP (80.2%, 89.5%, 71.2%, 93.3% and 87.2% respectively) (p < 0.05 for all). As compared to iDose^4^, PPV (85.3% vs. 80.5%, p < 0.05) and accuracy (93.6% vs. 91.4%, p < 0.05) of IMR showed a significantly higher (Fig 3)

### Comparison of IMR performance between 100 kVp and 120 kVp groups

Table 5 shows the overall summary of image quality and diagnostic performance of IMR between 120 kVp and 100 kVp.

**Table 5:** Image quality and diagnostic performance of IMR between 100 kVp and 120 kVp protocol.

	100-kVp group (n = 30)	120-kVp group (n = 30)	p-value
***Objective image quality***			
***- Mean Attenuation (HU) of ROI***			
Ascending aorta	461.1 ± 68.4	415.2 ± 72.4	0.014
Pulmonary artery	393.1 ± 79.4	341.9 ± 74.6	0.013
Left ventricle cavity	450.8 ± 66.4	380.0 ± 66.9	<0.001*
Left ventricle septum	131.0 ± 28.1	106.4 ± 16.5	<0.001*
***- Image noise (SD) of ROI***			
Ascending aorta	17.4 ± 3.5	16.5 ± 2.6	0.297
Pulmonary artery	17.5 ± 3.8	16.6 ± 4.9	0.402
Left ventricle cavity	19.0 ± 4.1	15.4 ± 2.5	<0.001*
Left ventricle septum	19.1 ± 5.1	16.1 ± 3.2	0.009*
***Subjective image quality***			
LAD	3.67 ± 0.66	3.73 ± 0.45	0.650
LCx	3.55 ± 0.69	3.80 ± 0.41	0.112
RCA	3.57 ± 0.68	3.78 ± 0.42	0.115
Total	3.66 ± 0.60	3.77 ± 0.43	0.155
***Diagnostic accuracy***			
***- per patient***			
Sensitivity	96.2%	100%	NS
Specificity	75.0%	60.0%	NS
Positive predictive value	96.2%	92.6%	NS
Negative predictive value	75.0%	100%	NS
Diagnostic accuracy	93.3%	93.3%	1.0
***- per segment***			
Sensitivity	89.2%	90.3%	0.964
Specificity	95.0%	95.6%	0.856
Positive predictive value	85.3%	87.2%	0.994
Negative predictive value	96.4%	96.8%	0.827
Diagnostic accuracy	93.6%	94.3%	0.768

Data are means ± standard deviations of the visualization score.

Note–HU, Hounsefield unit; ROI, region of interest; SD, standard deviation; LAD, left anterior descending artery; LCX, left circumflex artery; RCA, right coronary artery

* p <0.05

In terms of objective image quality, the mean attenuations at all locations in the 100kVp group were all significantly higher than those in the 120 kVp group (all p < 0.05). However, the noise in the ascending aorta and pulmonary artery were not significantly different between the two groups, whereas the noise of LV cavity (19.0 ± 4.1 vs. 15.4 ± 2.5; p < 0.001) and septum (19.1 ± 5.1 vs. 16.1 ± 3.2; p = 0.009) were significantly lower in the 120 kVp group. There were no significant differences in the subjective coronary image quality between the two groups (3.66 ± 0.60 (100 kVp) vs. 3.77 ± 0.43 (120 kVp); p = 0.155).

For comparison of diagnostic performance of IMR between 100 kVp and 120 kVp, there were no significant differences in both per-patient and per-segment analyses (all p> 0.05).

## Discussion

Our study revealed that it may be useful to use IMR with 100kVp protocol to assess heavily calcified coronary vessels. A comparison of three different reconstruction techniques in any kVp protocols revealed that IMR had better subjective and objective image qualities than FBP and iDose^4^, as well as a significantly improved diagnostic performance compared with FBP. While the diagnostic performance of IMR was comparable to that of iDose^4^, IMR showed significant improvements with respect to PPV and accuracy. We also showed that there were no significant differences in the overall image quality and diagnostic performance of IMR between the 100 kVp group and the 120 kVp group, although image noise in some structures (LV cavity and septum) was observed to be marginally higher in the 100-kVp group.

In the era of widespread use of CT, lowering the radiation dose may especially be an important consideration for patients requiring frequent follow-ups or for pediatric patients. However, lowering the radiation dose leads to increased noise. Therefore, noise-reducing IR algorithms, which can improve image quality and provide a similar diagnostic value compared with routine-dose FBP, have recently been developed and applied for both cardiopulmonary and body imaging [10]. Further advancements to the iterative reconstruction approaches have resulted in sophisticated knowledge-based iterative reconstruction algorithms, such as IMR. Several studies reported that IMR has been shown to significantly improve image quality, while at the same time reduce noise and artifacts compared with FBP and hybrid-IR [10, 15]. Our study supplements these investigations demonstrating superior image quality with reduced noise using a tube energy of 100 kVp. These results are similar with previous studies by Yuki et al. [16] and Oda et al. [15].

Although a lot of investigations focused on reducing radiation dose while preserving image quality, there is limited work investigating the utility of IMR for the evaluation of calcified coronary vessels. Regardless of CT technical development, traditional FBP has been identified to have limited diagnostic performance in the presence of heavy coronary artery calcifications [17, 18]. Moreover, to reduce blooming artifacts, it is common to use high kVp imaging, besides thin-section thickness, or sharp convolution kernels [6, 19]. When evaluating dense calcified coronary arteries, therefore, there has been a dilemma as to which of the radiation dose and image quality should be prioritized. In this regard, IMR can provide the possibility of decreasing calcium-related noise because it has the ability to decouple spatial resolution and image noise, while offering the potential to selectively improve high contrast resolution without affecting image noise in low-contrast areas [20, 21]. Renker et al. [9] using equipment of a different vendor showed that the use of iterative reconstruction (IR) reduces image noise and blooming artifacts, leading to improved diagnostic accuracy for calcified coronary vessels. However, 80% of patients used in their study were scanned using 120 kVp. Recently, Karolyi et al. [22] also reported that the use of IMR can improve image quality of CCTA and decreased calcified coronary plaque volumes. They also performed CCTA using 120 kVp protocols. However, our study included patients with heavily calcified coronary vessels, typically with scores ≥ 400 (mean Agatston score: 1308.71), and with CCTA performed using 100kVp as well as 120 kVp. Our results show that IMR used in combination with a lower kVp can improve diagnostic accuracy over traditional FBP or hybrid-IR, while maintaining similar diagnostic accuracy to 120-kVp for the evaluation of heavily calcified coronary vessels.

In general, increasing the average x-ray energy reduces calcium blooming, the image quality or diagnostic accuracy was expected to be high with higher radiation dose. However, our study shows that the image quality–both subjective and objective–and diagnostic accuracy of heavily calcified vessels can be maintained with the use of IMR at lower tube energies. Although some structures (LV cavity and septum) have been reported to have a marginally higher image noise in the low-kVp group, their effect on the diagnostic accuracy of CAD is not significant.

Our study has several limitations. First, due to the retrospective and nonrandomized design, there may be selection bias. Second, the study population was small (n = 60), which could weaken statistical power. Third, the mean effective dose was relatively high because we used a retrospective ECG-gating protocol in all patients due to the need for cardiac functional analysis.

In conclusion, 100 kVp IMR may be useful for assessing heavily calcified coronary vessels. It was shown to provide better diagnostic accuracy than FBP or iDose^4^ at the same 100 kVp, while maintaining similar diagnostic performance with the 120 kVp CCTA scans.
